# Posttraumatic stress disorder intervention for people with severe mental illness in a low-income country primary care setting: a randomized feasibility trial protocol

**DOI:** 10.1186/s40814-021-00883-3

**Published:** 2021-07-30

**Authors:** Lauren C. Ng, Eyerusalem Getachew Serba, Benyam W. Dubale, Abebaw Fekadu, Charlotte Hanlon

**Affiliations:** 1 Department of Psychology, University of California Los Angeles, Los Angeles, CA USA; 2 Department of Psychiatry, School of Medicine, College of Health Sciences, Addis Ababa University, Addis Ababa, Ethiopia; 3 Department of Psychiatry, University of Toronto, Toronto, ON Canada; 4 Centre for Innovative Drug Development and Therapeutic Trials for Africa (CDT-Africa), School of Medicine, College of Health Sciences, Addis Ababa University, Addis Ababa, Ethiopia; 5 Department of Global Health & Infection, Brighton and Sussex Medical School, Brighton, UK; 6 Centre for Global Mental Health, Health Service and Population Research Department, Institute of Psychiatry, Psychology & Neuroscience, King’s College London, London, UK; 7 Department of Psychiatry, WHO Collaborating Centre for Mental Health Research and Capacity-Building, School of Medicine, College of Health Sciences, Addis Ababa University, Addis Ababa, Ethiopia

**Keywords:** Global mental health, PTSD, Psychosis, Task-sharing, Intervention, Primary care

## Abstract

**Background:**

In this protocol, we outline a mixed-methods randomized feasibility trial of Brief Relaxation, Education and Trauma Healing (BREATHE) Ethiopia. BREATHE Ethiopia is a culturally and contextually adapted intervention for PTSD in participants with severe mental illness. BREATHE Ethiopia maps onto the World Health Organization’s guidelines for posttraumatic stress disorder (PTSD) treatment in low- and middle-income country primary care settings.

**Methods:**

Specifically, this study includes a non-randomized pre-pilot (*n* = 5) and a randomized feasibility trial comparing BREATHE Ethiopia to Treatment as Usual (*n* = 40) to assess trial procedures, acceptability, and feasibility of intervention delivery, and investigate potential effectiveness and implementation. In a process evaluation, we will collect data that will be critical for a future fully randomized controlled trial, including the numbers of participants who are eligible, who consent, who engage in treatment, and who complete the assessments, as well as the feasibility and acceptability of assessments and the intervention. Qualitative data on facilitators and barriers to intervention delivery and quantitative data on provider fidelity to the intervention and participant and provider satisfaction will also be collected. Quantitative assessments at baseline, post-treatment, 1-month follow-up, and 3-month follow-up will assess change in mental health symptoms and functional impairment and hypothesized intervention mechanisms, including knowledge about PTSD, stigma, trauma-related cognitions, and physiological arousal.

**Discussion:**

Findings from this study will inform a future fully-powered randomized controlled trial, and if found to be effective, the intervention has the potential to be integrated into mental healthcare scale-up efforts in other low-resource settings.

**Trial registration:**

Registered with ClinicalTrials.gov (NCT04385498) first posted May 13^th^, 2020; https://www.clinicaltrials.gov/ct2/show/NCT04385498?term=ethiopia&cond=PTSD&draw=2&rank=1.

**Supplementary Information:**

The online version contains supplementary material available at 10.1186/s40814-021-00883-3.

## Introduction

In most low- and middle-income countries (LMICs), severe mental illness (SMI), including psychotic and bipolar disorders, is a priority condition for treatment in primary care [1]. In high-income countries (HICs), 25–50% of people with SMI are thought to have posttraumatic stress disorder (PTSD) [2, 3]. Trauma exposure, particularly childhood abuse, has been identified as a risk factor for subsequent SMI [4–8], and SMI symptoms have been linked to increased risk of trauma [9, 10]. Moreover, SMI symptoms and associated treatment, including forced hospitalization and restraint, may also be distressing and traumatic events themselves [11–14].

For people with SMI, comorbid PTSD is associated with more severe SMI symptoms, greater functional impairment, and worse treatment outcomes [15–19]. Evidence-based interventions (EBIs) for PTSD in people with comorbid SMI are often effective in addressing PTSD symptoms [20–23]. However, these interventions were developed in HICs, and have not been evaluated in LMICs. Successful implementation of a psychotherapy intervention in Ethiopian primary care may provide a framework for interventions in other low-resource primary care settings, including those in HICs, where comorbid PTSD and SMI is usually overlooked, and integration of EBIs for comorbid PTSD and SMI remains minimal [10, 13, 24–32].

Despite the fact that PTSD may be higher in conflict-affected LMICs, and at least as prevalent in non-conflict LMIC settings, as compared to HICs [33–36], many LMICs have extremely limited mental health services making implementation of EBIs challenging [37, 38]. Low-income countries have less than one mental health provider per 100,000 people (compared to more than 50 in HICs), and almost all mental health services in low-income countries are provided in hospitals in large cities, with virtually no care available in rural areas [38–40]. Given the dearth of mental health specialists, primary care may be best positioned to address mental health in LMICs [41, 42], not just as an efficient means to address symptoms, but also because it provides care in the communities where people live, may be less stigmatizing, and has potential to integrate physical and mental health needs of people with SMI.

While integration of mental health services at the primary care level is critical, there are barriers that must be addressed [43]. In Ethiopia, like many LMICs, primary care clinics are staffed by providers with little training in mental health, limited time, and high staff turnover [43–47]. Rural populations also face barriers to care including lower literacy rates, large distances to clinics [43, 47, 48], and low awareness of, and high stigma related to, mental health concerns [49–51]. Despite these challenges, efforts are being made to implement evidence-based packages of care for people with SMI in Ethiopian primary care [44, 48, 52]. Although some interventions for PTSD have been adapted for use in LMICs, trials have excluded people with SMI [53–56]. Likewise, only a handful of psychosocial intervention studies for SMI have been conducted in LMICs, none of which assessed PTSD [57–60].

In this protocol, we describe a mixed methods randomized feasibility trial of Brief Relaxation, Education, and Trauma Healing (BREATHE) Ethiopia, a culturally and contextually adapted intervention for PTSD in patients with SMI [61]. BREATHE Ethiopia was selected for its brevity, its demonstrated effectiveness comparable to first line PTSD treatments, development for use with patients with comorbid SMI, and to address the pre-existing barriers in this low resource primary care setting, such as limited time and lack of staff with mental health training, that may preclude longer more complex treatments [62]. The aim is to test procedures for a fully powered trial and investigate implementation outcomes and potential effectiveness in a LMIC. Methods are discussed according to Standard Protocol Items: Recommendations for International Trials (SPIRIT) guidelines and all elements of the SPIRIT checklist are reported below [63].

## Method

### Study setting

The study setting is the primary health care (PHC) clinics and hospitals in and around Sodo and South Sodo districts. Selection of the health facilities will be based on an evaluation of the number of participants receiving mental health care, the accessibility of the clinic, the interest of the providers and facility leadership, and the other studies currently being run at each primary care facility. Sodo and South Sodo districts were the sites of the PRogramme for Improving Mental health care (PRIME) in Ethiopia [44]. This region is 90% rural and most of the 180,000 people live in villages widely spread apart and difficult to access. There is one primary hospital with general practitioner physicians, nurses, and health officers, and an outpatient psychiatric clinic run by a psychiatric nurse. There are also eight primary care clinics staffed by nurses and health officers. The number of staff per health center ranges from eight to 24, and there is high staff turnover. Approximately 20,000 to 40,000 people are served by each clinic.

PRIME was a large-scale, multi-country mental health services research program that investigated the implementation of evidence-based packages of mental health care integrated into primary care in Sodo District [44, 48, 52]. Although PRIME has concluded, the infrastructure that PRIME established will be leveraged for this project. PRIME has trained health extension workers (community health workers) to detect people with probable psychosis, including schizophrenia and bipolar disorder, and refer them to the local PHC facility. In that facility, primary care staff have been trained to carry out an assessment, make a diagnosis, prescribe psychotropic medication, provide psychoeducation, basic psychosocial care, support and monitoring, and refer if needed, for more intensive services to the primary hospital and to the specialty psychiatric hospital in Addis Ababa [44]. PRIME enrolled and delivered care to 300 patients with SMI, and helped train primary care staff, psychologists, and psychiatric nurses who might participate as interventionists or act as supervisors [44]. Finally, PRIME developed strong collaborations with local service users, caregivers, providers, and leaders, and has conducted formative research on stakeholder engagement, health system needs, and social, political and economic contexts [43, 44, 64].

### Selection and training of primary health care providers

Once primary care facilities have been identified, the local government leadership will be approached for assistance in identifying providers to train at each health center. Providers who have received mhGAP training and who are currently providing mental health services will be prioritized. Providers who are not selected for participation, but who also provide mental health services will be eligible to participate as treatment as usual (TAU) providers.

Using participatory methods, case scenarios, role plays, and video clips, we will train primary care staff and supervisors to deliver the adapted intervention. Training will be delivered through a multi-component package involving feedback, consultation, and supervision. We anticipate that training will be 5 days and will involve extensive role-play and case studies. Given current ongoing restrictions, training will be conducted virtually.

Before and after the 5-day training and after the pre-pilot, providers will be rated on their clinical skills using a standardized participant role play and will be evaluated by the PI of the study, and clinical supervisors using a tool (see Appendix 1) created for this intervention on their fidelity to the intervention and their skill in delivering the hypothesized active ingredients of the intervention including (1) breathing retraining, (2) psychoeducation, (3) positive coping, and (4) homework assignment and review. In addition, they will also be assessed on common therapeutic elements such as reflective listening and rapport building using the Enhancing Assessment of Common Therapeutic factors (ENACT) measure [65] and their knowledge of trauma and PTSD using an adapted version of the PTSD Knowledge Test, which has been used previously to assess change in PTSD knowledge after the BREATHE intervention [66]. Final provider selection for both the pre-pilot and the randomized feasibility trial will be based on provider interest and motivation, and the results of the post-training proficiency testing and ENACT and PTSD Knowledge test ratings.

### Supervision of the primary care providers

Ongoing supervision, consultation, and coaching during the pilot will be conducted using a “supervise the supervisors” model. Supervisors will be Ethiopian clinical psychologists who have experience working with individuals with SMI. LN will provide weekly virtual supervision to the supervisors over the phone, who will in turn provide weekly and as-needed supervision to the health center staff in person or over the phone. All supervision sessions will be audiotaped and coded to identify themes discussed and potential challenges and strengths of the intervention.

In order to facilitate rapid understanding of the intervention and to improve training and supervision of providers, participants in the pre-pilot will be asked to complete treatment sessions twice a week and to commit to completing all five sessions in 2.5 weeks. During the pre-pilot, providers will receive supervision twice per week and supervisors will observe at least one session per provider in person and will complete ENACT ratings for every session they observe in person. In addition, all sessions will be audio recorded and will be rated for fidelity to the intervention (see Appendix 1) within 1 day of the session taking place. Supervisors and providers will receive fidelity ratings from previous sessions, to be discussed and problem solved during supervision.

During the randomized feasibility trial, intervention sessions will be audio recorded, and supervisors and primary care providers will review clips of the sessions during supervision. A random sample of 50% of sessions will be rated for fidelity to the intervention and fidelity ratings will be provided to supervisors and providers for discussion during supervision.

### Participant eligibility, screening, and consenting

**Fig. 1:**
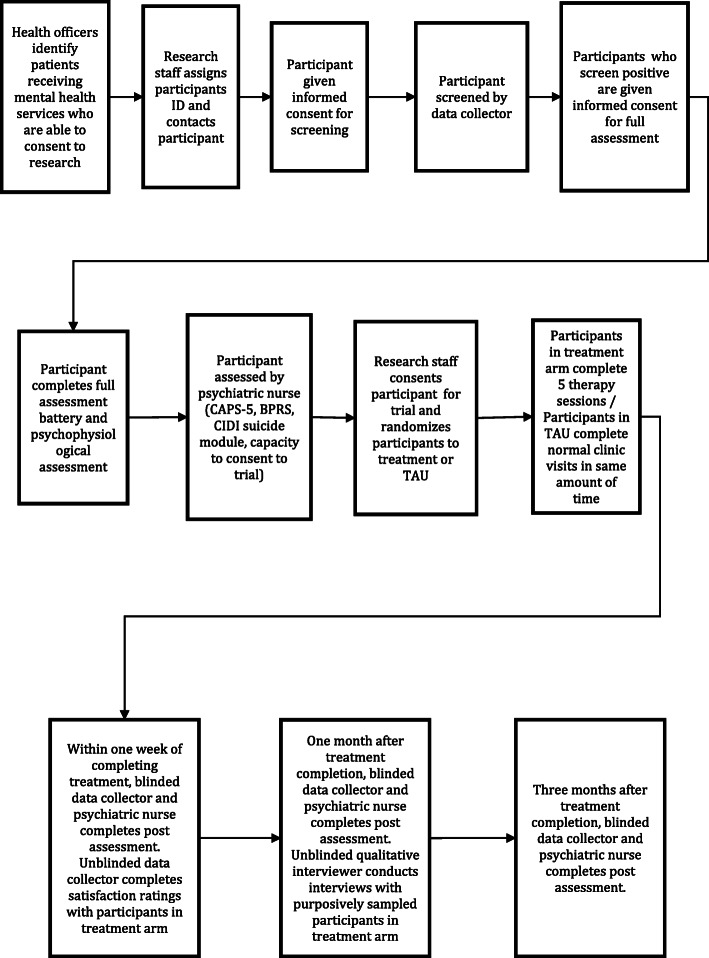
Steps of study design

See Fig. 1 for the steps of the pre-pilot and the randomized feasibility trial study design. Primary health care providers will be asked to provide a list of all the potential participants patients who meet the following inclusion criteria: (1) at least 18 years old; (2) able to complete procedures in Amharic; and (3) have met with a provider at the health facility for a mental health problem and/or received a psychotropic medication from the primary health facility in the last year. Providers will then be oriented to the concept of “consent for research.” For each identified patient, providers will select “Yes,” “No,” or “Don’t know” whether patients are cognitively able to consent for research. Patients who are rated “Yes” will be contacted by research staff to be given informed consent to participate in the screening study. Patients that are deemed “Don’t know” will be contacted to be assessed for the ability to consent by a psychiatric nurse using a capacity to consent rating form [67] and if they are rated by the nurse as being able to consent, they will be given informed consent. Patients who are rated “No” will not be contacted for participation. After patients are identified as being able to consent for research by health providers or the psychiatric nurse, if a phone number or home location is available, research staff will contact patients directly to see if they are interested in participating and being screened. If contact information is not available, patients will be identified and approached for participation as they come into the health facilities for clinical services. During the initial screening, eligible participants will be asked for preferred contact information.

Participants who provide informed consent to be screened for the study will be provided with 100 Ethiopian Birr (ETB; $2.70 USD) for participating in screening. Screening will occur in two steps. Step one of the screening involves participants providing self-report responses to the following measures administered by a data collector:

### Sociodemographics

The PRIME sociodemographic questionnaire will collect data on participant sex, age, education, marital status, family income, and food insecurity [52, 68].

### Trauma exposure

Trauma exposure will be assessed using an adapted version of the Life Events Checklist for DSM-5 [69] that includes items that were found to be relevant for the target population during qualitative studies (i.e., animal attack, drowning/near drowning, abduction for marriage without consent).

### Self-reported PTSD symptoms

PTSD symptoms will be assessed using the qualitatively adapted PTSD Checklist for DSM-5 [70] which includes suicidal ideation resulting from trauma exposure.

### Functional impairment

Functional impairment will be assessed using a 12-item version of the WHODAS-2 to assess functional impairment [71].

Participants who meet the following inclusion criteria will be given a second informed consent and will proceed to step two of the screening process: Identified as having (a) experienced a traumatic event; (b) associated PTSD symptoms defined as scores on the PTSD Checklist DSM-5 (PCL-5) [70] rated as 2 = “Moderately” or higher for at least four items—(we have a low threshold of symptoms because we do not know what the validated cut off or presentation of PTSD is in Ethiopia); and (c) any associated functional impairment on the WHODAS-2 (i.e., scores of 1 or higher on any item) [71].

Participants who meet criteria and consent for further screening for the trial will be provided with 100 ETB for further screening and will proceed to step two of the screening which involves being assessed by a psychiatric nurse for (a) *Capacity to Consent* to both the BREATHE intervention and to the research study using a rating scale to assess participant capacity to consent to the study [67]; (b) *Clinician*-*rated PTSD symptoms and diagnosis* using the past-month version of the Clinician Administered PTSD Scale for DSM-5 (CAPS-5) [72]; (c) *SMI symptoms* using the Brief Psychiatric Rating Scale Expanded Version 4.0 (BPRS-4.0) [73]; and (d) *Suicidal ideation* using the Suicidal Ideation module of the CIDI [74].

Participants who meet the following inclusion and exclusion criteria will be consented for participation in the pre-pilot or randomized feasibility trial: determined by the psychiatric nurse to (1) be cognitively and functionally capable of attending and participating in the therapy sessions; (2) be cognitively and functionally capable of providing informed consent for research and randomization; (3) be not currently high risk of suicide as measured by an endorsement of current or recent suicide plan or attempt on the CIDI Suicide module and psychiatric nurse clinical assessment of suicide risk; and (4) have a diagnosis of PTSD. Participants who are identified as having current high suicide risk will be followed up by primary care providers and receive referrals for higher levels of care, as needed. At any point in the study if patients exhibit distress and/or increasing severity of symptoms that warrants a higher level of care, they will be referred to the district hospital or to Butajira hospital or to Amanuel, the tertiary psychiatric hospital in Addis Ababa.

### Pre-pilot and intervention refinement

The first five participants who meet all inclusion criteria and consent to the study will receive the adapted intervention as part of the pre-pilot. Participants in the pre-pilot will be asked to complete treatment sessions twice a week and to commit to completing all five sessions in 2.5 weeks. To account for this intensive schedule, participants will be provided 100 ETB ($2.70 USD) for each treatment session and will be drawn from communities that are close to the health facilities. Each provider who will be delivering the treatment in the randomized feasibility trial will deliver the intervention to one participant in the pre-pilot phase (anticipated *n* = 5).

After all of the pre-pilot participants complete their post-treatment quantitative and qualitative assessments and satisfaction ratings, the quantitative results will be summarized. The audio recordings of the qualitative interviews will be analyzed using rapid assessment [75, 76] to facilitate efficient analysis and enable us to use results to improve the intervention and/or research processes before the start of the randomized feasibility pilot trial. The providers will participate in an audio-recorded focus group to understand their experiences during the pre-pilot and to gather data on ways that the intervention and research protocols could be improved.

In addition, two theory of change (ToC) workshops [77] will be convened, one with the providers and supervisors who participated in the pre-pilot, and one with the participants who participated in the pre-pilot. The ToC workshops will be used to understand the mechanisms of action in the intervention and to further refine the manual.

Any needed changes to the intervention or research protocol that were identified during the post-intervention qualitative interviews with participants, the focus group with providers, or either of the ToC workshops will be implemented prior to the start of participant enrollment for the randomized feasibility trial. The research team will complete the TIDieR checklist [78] to document any changes and their rationale that occurred after pre-piloting. A second TIDieR checklist will be completed after the feasibility trial is complete.

### Intervention

BREATHE Ethiopia is a treatment for post-traumatic stress disorder (PTSD) symptoms for use with individuals who have a diagnosis of PTSD or “probable PTSD.” This program is adapted from the “B.R.E.A.T.H.E.—Brief Relaxation, Education and Trauma HEaling: A Brief Intervention for Persons with PTSD and Co-Occurring Serious Mental Health Conditions” [79].

This intervention is designed to be delivered in primary care and fits within the Ethiopian Ministry of Health’s mandate to expand the care for people living with mental health conditions. Given the technical challenges in the field the intervention is designed to be delivered in person and has been adapted to follow mitigation procedures as per current ongoing restrictions. The program meets the guidelines provided by the World Health Organization’s Mental Health Gap Action Program (mhGAP) module on the Assessment and Management of Conditions Specifically Related to Stress [1]. The intervention was culturally and contextually adapted using qualitative semi-structured interview data from 48 participants including patients with SMI (*n* = 13); caregivers of patients with SMI (*n* = 13); health care providers (*n* = 13); and community and religious leaders (*n* = 9) from Sodo District, Ethiopia [80]. Adaptations were made to the intervention delivery and content.

Intervention delivery adaptations included changing (1) the session number and length from three 50-min sessions to five 20- to 30-min sessions, to accommodate the needs of the primary health care centers; and (2) the frequency of sessions from once a week to anywhere from once a week to once a month, to accommodate travel difficulties for patients and to map onto the normal frequency with which patients visit the primary care facilities for medication management follow-up visits. Content adaptations included (1) adding in positive coping skills and practices that were identified from the qualitative data and that were feasible in the rural Ethiopian context, such as purposeful engagement in positive activities; (2) changing the language to be more acceptable and understandable in Amharic (for example, changing “hyperarousal” to “over alertness”); (3) adding in psychoeducation about SMI symptoms and stigma; and (4) providing more guidance to providers on communication strategies, specifically ways to present the intervention in encouraging, normalizing, and non-stigmatizing ways.

The intervention consists of three main parts spread over five brief treatment sessions: (1) breathing retraining: participants are taught and encouraged to use breathing retraining to reduce current or anticipated physiological arousal that is associated with anxiety. Breathing retraining is taught in the first session and reviewed in all subsequent sessions; (2) normalizing through psychoeducation: the intervention teaches participants and (with participants’ permission) their families about the commonness of trauma, trauma symptoms, and related problems so that they understand that their reactions are normal and experienced by other people too. This information is presented using information handouts, worksheets, and discussion; and (3) positive coping: the intervention provides information about the relationship between PTSD symptoms and related problems and encourages participants to use positive coping strategies to help them feel better and more able to achieve their goals.

### Randomized pilot feasibility trial design

This study uses a type 1 hybrid effectiveness-implementation design [81] to conduct a randomized pilot feasibility trial (*n* = 40). The goal of the study is to assess trial procedures, refine the intervention and its implementation, and explore effectiveness and implementation outcomes. See Fig. 1 for steps of the study design.

### Quantitative assessments

We will gather preliminary data on demographics and SMI symptoms, effectiveness outcomes including change in hypothesized treatment mechanisms, and change in symptoms and functional impairment.

### Demographics and SMI symptoms

#### SMI symptoms

A psychiatric nurse will rate SMI symptom severity using the Brief Psychiatric Rating Scale Expanded Version 4.0 (BPRS-4.0) [73].

#### Clinical diagnosis and characteristics

Health providers will be asked to complete a short form about participant diagnosis, medication name, dose, and frequency as well as whether the participant has been adherent to medication during the last 3 months. Participant psychiatric diagnoses, medication, dates of attended mental health visits during the year prior to the baseline assessment and up through the 3-month follow-up, and length of psychiatric illness will be extracted from medical records at the health center.

### Treatment mechanisms

#### PTSD knowledge

Accurate knowledge about PTSD will be assessed using an adapted version of the PTSD Knowledge Test which has been used previously to assess change in PTSD knowledge after the BREATHE intervention [66]. The adapted PTSD Knowledge Test is a 14-item true/false and multiple-choice test that assesses information about trauma exposure, PTSD symptoms, associated problems, and treatment outcomes.

#### Mental illness-related stigma

Mental illness-related stigma will be measured by the 10-item version of the Internalized Stigma of Mental Illness Scale (ISMI-10) [82], which has been validated in Ethiopia [49, 83].

#### Post-trauma-related cognitions

Trauma-related cognitions will be assessed by the Post-Traumatic Cognitions Inventory [84] which is a 36-item measure assessing negative and inaccurate thoughts that are common in people with PTSD.

#### Self-reported arousal

The Self-Assessment Manikin (SAM) will be used to assess three domains of present-moment affective state: valence (e.g., positive or negative), arousal (e.g., calm or excited), and dominance (e.g., powerful or weak). Each affective domain is rated by participants on a 5-point scale using pictures [85].

#### Psychophysiological arousal

If found to be feasible, acceptable, and accurate following usability testing, physiological arousal will be measured by increases in heart rate variability [86, 87] using the Empatica E4 wristband wearable device [88] and Mindfield ESense Skin Response system [89] during the PhenX Toolkit’s trauma challenge assessment [90] which was adapted from Foa & Rothbaum’s Standardized Trauma Interview [91], in which participants will be asked to think about and recount details of their traumatic experiences. Similar to other trauma assessment questionnaires and interviews, the PhenX Toolkit’s trauma challenge assessment interview asks about a participants’ trauma experiences and assesses symptoms, while also assessing physiological arousal before during and after discussion of the trauma experiences and symptoms [90, 92]. At each assessment time point, data will be collected for a total of 15 min including 5 min during baseline, 5 min during the trauma interview, and 5 min during the trauma imagery.

### Use of stress management strategies

For participants in the intervention condition, use of stress management strategies that are taught during the intervention, including breathing retraining and positive coping skills, will be assessed by therapists during in-session homework review, and will be validated by fidelity raters when they review the audio-recorded sessions.

### Mental health symptoms and functional impairment

#### Suicidal ideation

Suicidal ideation will be assessed using the Suicidal Ideation module of the CIDI [74].

#### Alcohol misuse

Hazardous alcohol use will be assessed using the Fast Alcohol Screening Test (FAST) [93] adapted to include conversions for local alcoholic beverages [94].

### Clinician-rated PTSD symptoms and diagnosis

Psychiatric nurses will interview participants using the past-month version of the Clinician Administered PTSD Scale for DSM-5 (CAPS-5). Nurses will score participants to generate an overall symptom severity and cluster ratings. They will also use the score on the CAPS-5 to decide about whether or not participants meet current DSM-5 diagnostic criteria for PTSD.

### Self-reported PTSD symptoms

PTSD symptoms in reference to the initially identified traumatic event will be assessed using the Ethiopia adapted version of the PTSD Checklist for DSM-5 (PCL-5) [95].

### Functional impairment

Functional impairment will be assessed by the WHO Disability Assessment Schedule II (WHODAS 2.0) 12 item version, which has been used previously in Ethiopia [96, 97].

### Depression symptoms

Depression symptoms will be measured by an adapted version of the Patient Health Questionnaire (PHQ-9) [98] which has been adapted for and used in Ethiopia [99].

#### Intervention satisfaction

Participants and caregivers will complete satisfaction ratings that have been developed and validated in Ethiopia [47] during their post-assessment and providers will complete ratings of their perception of participant satisfaction, after the participant’s last treatment session.

### Feasibility trial assessment schedule

Quantitative assessments will be conducted by masked data collectors and psychiatric nurses at baseline, post-treatment: defined as the same day up to 1 week after the last session (post), 1-month after treatment completion (1-month follow-up), and 3-months after treatment completion (3-month follow-up; see Fig. 1 and Table 1). Intervention and healthcare satisfaction ratings will be completed by providers and caregivers (with participant consent) at post-assessment. Qualitative interviews with participants who will be purposively sampled based on those who do and do not complete all of the treatment sessions and those who do and do not have positive symptom improvement will be conducted during the 1-month follow-up by a different researcher to preserve blinding. Participants who do not want to continue therapy sessions may still participate in assessments.

**Table 1:** SPIRIT 2013 figure

	Screening	Enrolment	Allocation	Study period				
				Post-allocation				Close-out
**Timepoint**				**Sessions 1–5**	**+< 1 week**	**+ 1 month**	**+ 3 month**	
**Enrolment**								
**Eligibility screen**	X							
**Informed consent for Full Assessment**	X							
***Full Assessment screen***	X							
**Informed Consent for Trial**		X						
**Allocation**			X					
**Interventions**								
***BREATHE Ethiopia***				X				
***Treatment as usual***				X				
**Assessments**								
*Sociodemographics questionnaire*	X				X	X	X	
*Life Events Checklist for DSM-5*	X				X	X	X	
*adapted PTSD Checklist for DSM-5* (PCL-5)	X				X	X	X	
*WHO Disability Assessment Schedule II WHODAS-2 (12-item)*	X				X	X	X	
*Clinician Administered PTSD Scale for DSM-5 (CAPS-5)*		X			X	X	X	
*Brief Psychiatric Rating Scale Expanded Version 4.0 (BPRS-4.0)*		X			X	X	X	
*Suicidal Ideation module of the CIDI*		X			X	X	X	
*adapted PTSD Knowledge Test*		X			X	X	X	
*Internalized Stigma of Mental Illness Scale (ISMI-10)*		X			X	X	X	
*Post-Traumatic Cognitions Inventory*		X			X	X	X	
*The Self-Assessment Manikin (SAM)*		X			X	X	X	
*PhenX Toolkit’s trauma challenge assessment*		X			X	X	X	
*Use of Stress Management Strategies*		X		X	X	X	X	
*Fast Alcohol Screening Test (FAST)*		X			X	X	X	
*Patient Health Questionnaire (PHQ-9)*		X			X	X	X	
*Intervention and healthcare satisfaction ratings*					X			
*Qualitative interview*					X	X		

The time from initial contact to each assessment and each treatment session will be tracked. To allow for scheduling flexibility, participants will be able to complete each assessment up to 1 week before or after the target assessment date, for a total of a 2-week assessment window for the 1-month and 3-month follow-ups, and a 1-week assessment window for the post-assessment.

Since intervention participants may take between 5 weeks to 5 months to complete the intervention, participants in the TAU condition will be matched by study enrollment date and health center location to a participant in the intervention condition, and will complete follow-up dates on the same schedule. Since interview times may differ by participant, participants will be told they will complete five sessions and four interviews during the study, but will not be told the assessment schedule in advance.

Assessments will take place at the primary care center or hospital, unless this is not feasible for participants. If participants cannot come to the primary care center for assessments, then the assessments will be completed in participant’s homes or another location of their choice. All intervention and TAU sessions will take place in the health care center, and all participants will continue concomitant medication management and follow-up care.

When participants are initially consented for the trial, participants will be given a phone number and a 25 Ethiopian Birr (ETB) phone card to use to contact researchers about scheduling or questions or if they experience any adverse events. In addition, they will be asked how they would like to be contacted in the event that they have missed a session. Options will include a phone call or home visit by a researcher or no contact. If participants select a phone call or home visit, providers will attempt to contact them three times before discontinuing them from the study.

### Participation and retention

We will collect data on participant and provider participation and retention. Participant participation is defined as the number of eligible participants (i.e., met inclusion criteria for screening one and two) who agreed to participate in the feasibility trial. Participant retention is the number of participants who were randomized to the intervention condition who completed intervention sessions one, two, three, four, and/or five, as well as the number of all participants in the feasibility trial who completed the post-assessment, 1-month follow-up, and 3-month follow-up. Provider participation includes the number of providers who agreed to participate out of the total number who were offered the opportunity to participate. In addition, we will gather data on the number of providers trained to deliver the intervention, and the number who ultimately were rated highly enough on their clinical skills and knowledge of trauma and PTSD to be eligible to be providers during the pre-pilot and pilot. In addition, when participants, caregivers, and staff are offered or trained in the intervention but decline to participate, they will be asked to complete a brief interview assessing reasons for non-participation.

Quantitative data will be entered using REDCap software with smartphones and uploaded to a secure encrypted server. Data will be downloaded to a password protected and encrypted computers at UCLA facilities and Ethiopia field site. All paper data files and audio recorders will be kept securely stored in a locked file cabinet in a locked office at field site. Qualitative data and audio recordings will be uploaded to secure encrypted server and downloaded to a password protected and encrypted computer. Data management reports across the three following domains will be employed: entered, verified, and edited. These reports of data records will be evaluated once a month.

Ongoing supervision will be achieved through weekly phone calls and at least three times per year annual site visits, virtual or neighboring site personnel, to provide training and supervision. Study coordinators will ensure consistency of procedures, problems/challenges, and general training issues. For example, will address issues of data collection, budget, recruitment, data management and analysis, and perform training and ongoing review of the protocol and assessment procedures. Data will be examined weekly and feedback provided to research staff to make any corrections in as close to real time as possible.

### Randomization

Starting with the sixth participant enrolled (the first five participants will be in the pre-pilot), participants will be randomized to receive the intervention or treatment as usual (TAU) which includes medication management and follow-up at the health facilities. There are currently no other psychotherapies available so TAU was chosen as the comparator. The sixth to the 50^th^ participant (45 participants to be randomized to account for potential dropout) will be randomly assigned with a 1:1 allocation using a computer-generated randomization schedule that will be created by the PI. After screening is complete, researchers will run a randomization computer program to identify the assigned treatment arm. Researchers will then record the participant ID number and treatment arm in the study log and will also record the participant’s treatment arm in their medical chart. Researchers will then notify the health provider of the participant’s allocation and the date and time the participant will start the treatment or TAU. Participants, research staff, and psychiatric nurses conducting the pre, post, and follow-up assessment will be masked to the participant’s condition.

### Quantitative data analyses

With a small sample size, the analyses will be descriptive and exploratory and will primarily be used to inform a future fully powered trial. We will calculate descriptive statistics to assess participation, retention, satisfaction, and fidelity. We will explore the relationship between covariates and baseline outcome scores and retention, satisfaction, and fidelity to identify any characteristics that might be associated with higher or lower implementation success. In addition, we will calculate summary statistics on all treatment mechanisms, mental health outcomes, and covariates of interest. We will investigate how scores on the outcome measures listed above change over time, paying particular attention to change between baseline and post-intervention scores and comparison between the intervention and the TAU groups. We will conduct bivariate analyses to assess the association between covariates and outcomes. Participant and caregiver reports will be analyzed separately. For continuous outcomes, we will use mixed-models with the participant as the random intercept to assess repeated measures change over time. This approach was selected because it models all available data regardless of whether individuals were missed at some time points. If deemed necessary, we will employ variable transformation techniques to improve the normality assumption. The analysis will include all participants who completed the baseline assessment and at least one treatment session, effects of outlier data inclusion in analyses will be assessed, and if necessary missing data will be handled using multiple imputation. Given the aim of the study to assess feasibility and implementation and the low sample size, differences in comorbidities will not be a main focus of analysis. Future larger iterations of the protocol will take these into account.

### Qualitative interviews

To understand participant and provider experiences with the intervention and to identify and explain positive or negative treatment mechanisms or effects, we will conduct semi-structured interviews with all of the participants enrolled in the pre-pilot (*n* = 5), a purposive sample of half of the participants enrolled in the treatment arm of the feasibility trial (*n* = 13), and one interview with each primary health care providers who administered the intervention and their supervisors. The primary questions of interest are as follows: (1) what are the facilitators and barriers to delivering and sustaining the intervention and (2) how could the intervention be modified to improve acceptability, implementation, and sustainability? Interviews with participants will take place during the 1-month follow-up assessments, after the quantitative questionnaire. Participants will be purposively sampled to have approximately equal numbers of men and women, and to have equal numbers of participants who did well in the treatment and who did not do as well (based on satisfaction ratings and outcome measures completed during the post-assessment). In addition, we will purposively sample participants who dropped out of the treatment.

Interviews with healthcare providers and supervisors will occur after they have finished seeing their last participant. Questions of interest include experiences with the intervention and aspects of study design including randomization, logistical or other challenges with implementation, and emotional or behavioral changes during and after the intervention. In addition, we will ask participants for feedback on the feasibility and time burden of the assessments, audio recordings, screening, and other aspects of the research trial protocol. The interview guides include open-ended questions to facilitate inductive analyses and more specific probes related to a priori research questions.

All interviews and assessments will be conducted by trained researchers with experience interviewing participants with SMI, their caregivers, and community members. We will also conduct additional training on qualitative interviewing, study protocols and interview guides, and questions or concerns that might arise when asking about trauma or PTSD. Audio-recorded interviews will be transcribed and translated verbatim from Amharic into English. Each week, we will review the transcripts with the interviewers to discuss emerging themes, modify the interview guide as needed, and assess theoretical saturation.

### Qualitative data analytic plan

Analysis will be iterative and follow descriptive qualitative thematic content analysis [100, 101]. We will use a “framework analysis” [102] approach to facilitate analyses related to the central research questions. Concepts will be used to develop a codebook consisting of a label, a definition, and illustrative quotes from the data. We will review the coded transcripts to determine emerging themes. Final themes will be agreed upon in consultation with mentors and research staff. A research staff member will also code the transcripts and we will assess inter-rater reliability. Data will be re-examined in ongoing discussions to allow for further theorizing and making connections between research questions, coding categories, and raw data. We will explore links between emerging themes to guide intervention development and the implementation approach.

## Discussion

Globally, mental and substance use disorders account for 23% of years lost to disability, making them the leading cause of disability [103]. Areas of the world experiencing rapid population growth, such as sub-Saharan Africa, are estimated to have a 130% increase in the burden of disability due to mental disorders by 2050 [104]. In contrast to the massive need, in low-income countries, the estimated gap between those who need mental health care and those who receive it exceeds 90% [105–107].

In Ethiopia, more than 1% of the population [108] lives with SMI and 10% have at least one relative with SMI [109]. People with SMI in Ethiopia experience high rates of stigma, neglect, chaining and restraint, human rights abuses, physical and sexual violence, and road traffic accidents [44, 49, 51, 83, 109–117] and have been found to be victims of violence at higher rates than people without SMI [117]. Overall, 25% of people with SMI in Ethiopia die from unnatural causes [118]. In the general population, almost half of rural Ethiopians experienced major threatening events in the previous 6 months [52]. Providing primary health services that address trauma exposure and subsequent symptoms may reduce the negative impact of stressful life events and be acceptable and feasible in a low-resource setting. Indeed, service users, caregivers, and providers in Ethiopia support provision of mental health services in primary care due to ease of access, lower costs, and reduction in caregiver burden [119], and over 90% of primary health care workers in Ethiopia believe mental illness is a problem and want mental health services integrated into their health facilities [45, 119]. The Ethiopian government has made integration of mental health services into primary care a policy priority [39].

The current study uses mixed methods to evaluate the potential effectiveness and implementation of a psychotherapy intervention to treat PTSD in patients with SMI in Ethiopian primary care clinics.

Although some interventions for PTSD have been adapted for use in LMICs, trials have excluded people with SMI [53–56]. Likewise, only a handful of psychosocial intervention studies for SMI have been conducted in LMICs, none of which assessed PTSD [57–60]. Understanding whether interventions are feasible and effective in LMICs has implications for improving access to, and sustainability of, care, and has been identified as a critical research need [120] and a National Institute of Mental Health grand challenge in global mental health [121]. Moreover, successful implementation of a psychotherapy intervention in Ethiopian primary care may provide support and a framework for interventions in high-income countries, where comorbid PTSD and SMI is still usually overlooked, and integration of EBIs for comorbid PTSD and SMI remains minimal [10, 13, 24–32].

One of the central design considerations for this study was the selection of the BREATHE intervention [61, 62] for adaptation and implementation. Although first-line PTSD treatments such as CPT and PE have the largest evidence-base for PTSD treatment, trials have generally excluded people with SMI, and the interventions are highly intensive, leading to implementation challenges [122–125]. Since the goal of this study was to develop an intervention for PTSD symptoms for people with comorbid SMI in a low-resource setting with pre-existing barriers to implementation including no staff with mental health training and very limited time to spend with patients [43, 45–47, 126], we chose to use the BREATHE intervention which was developed for people with comorbid SMI, is brief and relatively simple, and has a strong theoretical basis and demonstrated effectiveness that is almost comparable to intensive first-line PTSD interventions [61, 127]. Indeed, assessing the feasibility, effectiveness, and implementation of the BREATHE intervention in a low-resource primary care setting has implications for PTSD treatment in the USA, as researchers have called for brief PTSD care models that can be easily implemented and sustained in real-world care [122].

Since the intervention is being developed for a new context, we decided to devote most of the research resources to a comprehensive qualitative and mixed methods approach to develop an in-depth understanding of the cultural and contextual factors that would facilitate or hinder effective delivery of the intervention rather than to a fully powered randomized trial to assess effectiveness. If the intervention is acceptable to participants and providers and implementation challenges can be addressed, we hope to test its effectiveness in a fully-powered randomized trial in the future.

In addition, we decided to utilize a hybrid effectiveness-implementation design rather than only focusing on effectiveness because the lack of available mental health services in Ethiopia and other low-income countries necessitates research designs that can rapidly move interventions from the laboratory to real-world clinical care. While an effectiveness design would allow for direct assessment of the intervention effect, the intervention might still not be suitable for the Ethiopian health care system. We believe that the type 1 hybrid effectiveness-implementation design [81] is the best model to develop an effective and contextually and culturally appropriate PTSD intervention that can be sustained in Ethiopian primary care.

Finally, for logistical purposes and the fact that health services are ordinarily offered in Amharic, we have limited participants to those who are able to complete procedures in Amharic. Although most people in Sodo and South Sodo districts are fluent in Amharic, this is not true of everyone, particularly those with very limited education. In addition, the first language of the region is Gurage, and so the intervention may not be as effective or acceptable if it is delivered in Amharic.

### Trial status

This is protocol version #5.0; July 25^th^, 2020 and this trial has not yet started recruitment.

## Supplementary Information

**Additional file 1.**

**Additional file 2.** Consent Form

## Data Availability

Not applicable. When complete, de-identified data generated during this study will be made available from the corresponding author on reasonable request.

## Abbreviations

LMICs: Low- and middle-income countries
SMI: Severe mental illness
HICs: High-income countries
PTSD: Posttraumatic stress disorder
EBIs: Evidence-based interventions
BREATHE: Brief Relaxation, Education and Trauma Healing
PHC: Primary health care
PRIME: PRogramme for Improving Mental health care
mhGAP: Mental Health Gap Action Program
TAU: Treatment as usual
ENACT: Enhancing Assessment of Common Therapeutic factors
ETB: Ethiopian Birr
ToC: Theory of change
