# Supplementary material for: Posttraumatic stress disorder intervention for people with severe mental illness in a low-income country primary care setting: a randomized feasibility trial protocol
Source: Pilot Feasibility Stud. 2021 Jul 30;7:149. doi: 10.1186/s40814-021-00883-3 (PMC8323310; doi:10.1186/s40814-021-00883-3)
Supplement: Supplementary file 1 — Additional file 1. [file 40814_2021_883_MOESM1_ESM.docx]

**Additional file 1**

| **Fidelity Checklist** | 1 = Poor,2 = Satisfactory, 3 = Excellent, 0 = Not Completed, N/a= Not Applicable | | |
| --- | --- | --- | --- |
| **ID#-Session#** |  | Rater |  |
| **Agenda Setting** |  |  |  |
| Articulate Specific Agenda |  |  |  |
| Identify Any other issues |  |  |  |
| Implement specific agenda |  |  |  |
| **Homework Review** |  |  |  |
| Review prior homework |  |  |  |
| Praise all efforts |  |  |  |
| Troubleshoot obstacles |  |  |  |
| **Overview of Program** |  |  |  |
| Focus of treatment program |  |  |  |
| Length of program & logistics |  |  |  |
| Description of treatment components |  |  |  |
| **Breathing Retraining** |  |  |  |
| Rationale |  |  |  |
| Explanation |  |  |  |
| Demonstration |  |  |  |
| Practice by client |  |  |  |
| **Use of Educational Materials** |  |  |  |
| Utilize handouts & worksheets |  |  |  |
| Distribute & review materials |  |  |  |
| Elicit & answer questions |  |  |  |
| **Psychoeducation** |  |  |  |
| Information about trauma & PTSD |  |  |  |
| Information about associated symptoms |  |  |  |
| Link patient symptoms to trauma/PTSD/ provide rationale |  |  |  |
| Elicit client’s symptoms |  |  |  |
| Answer questions |  |  |  |
| **Assign Homework** |  |  |  |
| Develop homework assignment |  |  |  |
| Collaborate with Client |  |  |  |
| Make specific plan |  |  |  |
| Troubleshoot obstacles |  |  |  |
| **Teaching Effectiveness** |  |  |  |
| Instill motivation to learn information & skills (e.g. coping, breathing, Sx management) |  |  |  |
| Teach information & skills (e.g. coping, breathing, Sx management) |  |  |  |
| Modeling/practice of skills (e.g. coping, breathing, Sx management) |  |  |  |
| Adaptation of skills as needed |  |  |  |
| Reinforcement of small steps/shaping |  |  |  |
| Encouragement |  |  |  |
| Use significant others (i.e. parents) to facilitate learning |  |  |  |
| **Interpersonal Effectiveness** |  |  |  |
| Facilitate communication (empathic nature) |  |  |  |
| Use client’s own language & phrases |  |  |  |
| Warm/Confidential/Professional |  |  |  |
| Provision of hope |  |  |  |
| **Pacing and Efficient Use of time** |  |  |  |
| Session length maximum 1 hour |  |  |  |
| Efficient structuring of time |  |  |  |
| Tactful limiting of peripheral & unproductive discussion |  |  |  |
| **Reduction of Client Distress (distress, stress, discomfort)** |  |  |  |
| Identify & respond to client distress |  |  |  |
| Empathy to show understanding |  |  |  |
| Use of education/skills to reduce distress |  |  |  |
| Make plan to address persistent distress |  |  |  |
| **Use of Positive Coping** |  |  |  |
| Discussed and Identified ways client can engage in positive coping |  |  |  |
| Clients engaged in positive coping at home |  |  |  |
| **Manual Adherence** |  |  |  |
| Followed individualized treatment for client |  |  |  |
| General adherence to manual guidelines & rules |  |  |  |
| **Use of brief Motivational Intervention** |  |  |  |
| Respond to client ambivalence about treatment with intervention |  |  |  |
| Follow manualized instructions for values-based treatment |  |  |  |
| Make plan for continuing treatment based on client's responses |  |  |  |
| Use client-centered language & promote autonomy |  |  |  |
| **Patient Engagement** |  |  |  |
| Patient actively participated during session/volunteered information |  |  |  |
| Patient completed/attempted to complete homework |  |  |  |
| Patient was open to new concepts/was willing to try them |  |  |  |
| **Overall Session Quality (Rate 1-5)** |  |  |  |
