# Supplementary material for: Posttraumatic stress disorder intervention for people with severe mental illness in a low-income country primary care setting: a randomized feasibility trial protocol
Source: Pilot Feasibility Stud. 2021 Jul 30;7:149. doi: 10.1186/s40814-021-00883-3 (PMC8323310; doi:10.1186/s40814-021-00883-3)
Supplement: Supplementary file 2 — Additional file 2. Consent Form [file 40814_2021_883_MOESM2_ESM.docx]

**Additional file 2**

Consent Form

CONSENT FORM FOR PTSD RANDOMIZED TRIAL

**Title of Study: Evaluation of PTSD Treatment**

Thank you for considering taking part in this research. The person organizing the research must explain the project to you before you agree to take part. If you have any questions, please ask the researcher before you decide whether to join in. You will be given a copy of this form to keep and refer to at any time.

**Principal Investigator:** Abebaw Fekadu, MD, PhD, Addis Ababa University

**Invitation Paragraph**

I would like to invite you to participate in this research project that is being conducted by faculty members in the departments of Psychiatry at Addis Ababa University and Boston University in the US. You should only participate if you want to; choosing not to take part will not disadvantage you in anyway and will not impact the care you receive or deliver at the health center.

Before you decide whether you want to take part, it is important for you to understand why the research is being done and what your participation will involve. Please take time to read or listen to the following information carefully and discuss it with others if you wish. Please ask me if there is anything that is not clear or if you would like more information.

**What is the purpose of the study?**

The aim of this study is to evaluate treatment for PTSD in the Sodo primary care centers. The study will involve four interviews spread out over several months with one of our trained researchers. Half of the participants will be randomly selected to receive a new treatment for PTSD at the health center. You will be asked to practice what you learn in between sessions.

**Why have I been invited to take part?**

We are inviting patients who have experienced a difficult event and are having negative thoughts and feelings about that event to take part in this study. You will be excluded if you are under the age of 18, cannot provide informed consent, or cannot complete the interview in Amharic or English. You will also be excluded and referred for additional services if you report that you have hurt yourself in the past month or that you may hurt yourself.

**Do I have to take part?**

Participation is voluntary. You do not have to take part. Please feel free to ask any questions about this study at any time during or after the review of this information sheet.

**What will happen to me if I take part?**

If you decide to take part you will be given this information to keep and will be asked to sign and initial the form. I will then discuss the interview procedure with you and arrange to interview you in a private place (for confidentiality reasons) or at a suitable venue in a local public site if you prefer.

The interview will take approximately 60 minutes and will be based on the interview topic guide. I will record your responses to the questions. With your permission, we would also like to interview a close family member or friend of your choice. After the interview you will be randomized to receive the new treatment or the usual treatment. You will be asked to come to the health center five times to learn new information and skills from the primary care providers. With your permission we will audio record these five treatment sessions. Regardless of the randomization you will still receive your medicine and all of your normal care. Everyone who participates will be reinterviewed three more times, for a total of four interviews.

Even if you have decided to take part, you are still free to stop your participation at any time during the interview and to have research data/information relating to you withdrawn without giving any reason up to three months after the interview.

**What are the potential risks?**

The main disadvantage to taking part in the study is that you may find some of the questions asked to be emotionally upsetting or uncomfortable. You might also feel tired during the interviews. Please let us know at any time if you would like to take a break or stop the interview. You may also refuse to answer any questions you choose. Participating or not participating will not impact you care or your employment in any way.

Because study visits involve meetings with research and/or clinical staff, there may be increased risk of exposure to Covid-19. To minimize this risk, all study staff and participants will be provided with, and required to use, face masks, and will be required to use hand sanitizer prior to any research or treatment visit or interaction. Whenever possible, there will be no more than one research staff member with each participant at any one time, with the exception of a patient’s caregiver, if needed. Everyone will be required to stand and/or sit 6 feet apart whenever possible, and all sessions will be conducted in well ventilated rooms or outside, as feasible and comfortable for participants and staff, while ensuring participant privacy. Research staff who feel ill or have a fever will not be allowed to work, and prior to study visits, all participants will be screened for the presence of covid-19 symptoms.

**What are the potential benefits?**

Your being in the study may help the investigators learn how to better understand how to improve the lives of people living with mental illness. Your thoughts on how to improve care for people with mental illness may impact the way treatment is delivered in the future. You may also benefit by having the opportunity to share your experiences, stories, opinions, and thoughts with the interviewer. However, you also may not receive any benefit.

**What are the costs?**

There are no costs to you for being in this research study.

**What is the compensation for this study?**

You and your family member/friend will each be compensated 100 ETB for each of the interviews that you participate in.

**Will my taking part be kept confidential?**

What is said in the interview is regarded as strictly confidential and will be held securely until the research is finished. All of the data will be anonymised. In reporting on the research findings, the names of any participants will not be revealed. All of the information gathered within the interviews will be held on password-locked computer files and locked file cabinets. If data needs to be transmitted between computers it will be sent securely through encrypted electronic communication or transported on encrypted flashdrives. No data will be accessed by anyone other than the research team. No data will be able to be linked back to any individual taking part in the interview.

**We will do our best to keep your information safe. However, we cannot guarantee confidentiality.**

We may reveal information outside of the research team is if we become concerned about your safety, the safety of someone else, or if we are concerned that you need medical attention. In that case we may need to contact a health care provider to help keep you safe.

Also, federal and state agencies, if they are required by law or are involved in research oversights, may access information about you from the study. Such agencies may include the US National Institutes of Health, Addis Ababa University Research Ethics Committee, or the Ethiopia National Research Ethics Review Committee.

**What is the alternative?**

Your alternative is to not participate in the study.

**How is the project being funded?**

The project is being funded by the United States’ National Institute of Mental Health. The study has been approved by the Addis Ababa University Research Ethics Committee and the Boston University Medical School Institutional Review Board.

**What will happen to the results of the study?**

A final report summarizing the main findings will be produced and will be sent to you. We also plan to disseminate the research findings through publication and conferences around the world.

**Who should I contact for further information?**

The investigator or a member of the research team will try to answer all of your questions. If you have any questions or require more information about this study, please call and speak to Dr. Abebaw Fekadu (tel. 0118962052).

**What if something goes wrong?**

If you have concerns about the ethical conduct of the study or any complaints, please speak to the Institutional Review Board of the College of Health Sciences, Addis Ababa University (tel. +251118961396; email: chs.irb@aau.edu.et ).

The Boston Medical Center/Boston University Medical Campus IRB have also reviewed this study. The IRB is a group that helps monitor research.

**What are my rights?**

By consenting to be in this study you do not waive any of your legal rights. Consenting means that you have been given information about his study and that you agree to participate in the study. You will be given a copy of this form to keep.

If you do not agree to be in this study or if at any time you withdraw from this study you will not suffer any penalty or lose any benefits to which you are entitled. Your participation is completely up to you. Your decision will not affect your ability to get health care or payment for your health care. It will not affect your enrollment in any health plan or benefits you can get.

**Thank you for your time and for considering taking part in this research.**

I confirm that I understand that by ticking/initialling each box I am consenting to this element of the study. I understand that it will be assumed that unticked/initialled boxes mean that I DO NOT consent to that part of the study. I understand that by not giving consent for any one element I may be deemed ineligible for the study.

**Please tick or initial**

1. I confirm that I have read and understood the information for the above study. I have had the opportunity to consider the information and asked questions that have been answered satisfactorily.
2. I understand that my participation is voluntary and that I am free to withdraw at any time without giving any reason. Furthermore, I understand that I will be able to withdraw my data up to 3 months after my interview
3. I consent to the processing of my personal information for the purposes explained to me.
4. I understand that my information may be subject to review by responsible individuals from Addis Ababa University and Boston University for monitoring and audit purposes.
5. I understand that confidentiality and anonymity will be maintained and it will not be possible to identify me in any publications
6. I agree to be contacted in the future by researchers who would like to invite me to participate in follow up studies to this project, or in future studies of a similar nature.
7. I agree that the research team may use my data for future research and understand that any such use of unidentifiable data would be reviewed and approved by a research ethics committee. In such cases, as with this project, data would not be identifiable in any report.
8. I agree to have my treatment sessions and interviews audio recorded.
9. I understand that the information I have submitted will be published as report.
10. I understand that I must not take part if I fall under the exclusion criteria as detailed in the information sheet and explained to me by the researcher.
11. I agree that if the researchers become concerned about my or someone else’s safety, they may stop the interview and withdraw me from the study.
12. I understand that confidentiality cannot be guaranteed if the interviewer is concerned about my safety or the safety of someone else.
13. I agree that health care providers may be contacted if the researchers become concerned about my safety or the safety of others.
14. I understand that I may ask any of the study staff to stop the interview and I may withdraw from the study at any time and there will be no negative consequences.
15. I understand that the risk to participants includes possible breach of confidentiality, interview fatigue, or personal discomfort with some of the questions.
16. I understand that I will be compensated 100 ETB for my time.
17. I agree that I do not waive any of my legal rights by agreeing to participate in this study.
18. I agree to use hand sanitizer prior to any research or treatment visit or interaction, and I agree to wear a face mask and maintain physical distancing during all research related visits and interactions.
19. I agree to answer questions about my exposure to Covid-19 and whether I have any Covid-19 symptoms prior to any study visits.

**__________________ __________________ _________________**

**Name of Participant Date Signature**

__________________ __________________ _________________

Name of Witness (if applicable) Date Signature

**__________________ __________________ _________________**

**Name of Researcher Date Signature**
